# Effects of different integrase strand transfer inhibitors on body weight in patients with HIV/AIDS: a network meta-analysis

**DOI:** 10.1186/s12879-022-07091-1

**Published:** 2022-02-03

**Authors:** Ruojing Bai, Shiyun Lv, Hao Wu, Lili Dai

**Affiliations:** 1 Center for Infectious Diseases, Beijing Youan Hospital, Capital Medical University, Beijing, China; 2 Travel Clinic, Center for Infectious Diseases, Beijing Youan Hospital, Capital Medical University, Beijing, 100069 China

**Keywords:** Integrase inhibitors, HIV/AIDS, Body weight, Network meta-analysis

## Abstract

**Background:**

Global antiretroviral therapy has entered a new era. Integrase strand transfer inhibitor (INSTI) has become the first choice in acquired immunodeficiency syndrome (AIDS) treatment. Because INSTI has high antiviral efficacy, rapid virus inhibition, and good tolerance. However, INSTIs may increase the risk of obesity. Each INSTI has its unique impact on weight gain in patients with human immunodeficiency virus (HIV)/AIDS. This study systematically assessed different INSTIs in causing significant weight gain in HIV/AIDS patients by integrating data from relevant literature.

**Methods:**

PubMed, Embase, Cochrane Library, China National Knowledge Infrastructure (CNKI), Chinese Biomedical Literature Database (CBM), China Science and Technology Journal Database (VIP), and Wanfang databases were searched to find studies on the influence of different INSTIs in weight gain. Data on weight change were extracted, and a network meta-analysis was performed.

**Results:**

Eight studies reported weight changes in HIV/AIDS patients were included. Results of the network meta-analysis showed that the weight gain of HIV/AIDS patients treated with Dolutegravir (DTG) was significantly higher than that of Elvitegravir (EVG) [MD = 1.13, (0.18–2.07)]. The consistency test results showed no overall and local inconsistency, and no significant difference in the results of the direct and indirect comparison was detected (*p* > 0.05). The rank order of probability was DTG (79.2%) > Bictegravir (BIC) (77.9%) > Raltegravir (RAL) (33.2%) > EVG (9.7%), suggesting that DTG may be the INSTI drug that causes the most significant weight gain in HIV/AIDS patients.

**Conclusion:**

According to the data analysis, among the existing INSTIs, DTG may be the drug that causes the most significant weight gain in HIV/AIDS patients, followed by BIC.

**Supplementary Information:**

The online version contains supplementary material available at 10.1186/s12879-022-07091-1.

## Background

Acquired immune deficiency syndrome (AIDS) refers to an infectious disease with great hazards, and it is caused by infection of the human immunodeficiency virus (HIV). HIV is a virus that could attack the human immune system. This virus would primarily target CD4^+^ T lymphocytes, which are the most prominent cells in the human immune system, and destroy them in large quantities, thereby causing the human body to lose its immune function [1, 2]. This makes a patient with HIV infection susceptible to various diseases, and the infection also increases the incidence of malignant tumors [3, 4]. Yet, mortality of HIV-infected or AIDS patients decreases significantly with timely treatment using antiretroviral therapy (ART)[5]. Integrase strand transfer inhibitors (INSTI), a new class of antiviral drugs, includes Dolutegravir (DTG), Raltegravir (RAL), Elvitegravir (EVG), and Bictegravir (BIC), have good efficacy and tolerability [6–8]. Multiple guidelines have recommended these drugs for treating HIV/AIDS [9–12]. Yet, some studies [13, 14] found that patients treated with INSTI had more significant weight gain than patients who used conventional antiviral therapy (without INSTI).

During the first two years of ART treatment, significant weight gain has become a recognized problem for the patients [15, 16]. In the early course of treatment, weight gain is an important sign of recovery, indicating the restoration of immunity and the improvement of the survival rate in the patients [17–21]. However, some studies [22, 23] found that more than half of the HIV/AIDS patients who received ART for up to 3 years were overweight or obese, and the potential impacts of weight gain in HIV/AIDS patients are not clear. In general, the obese population has a significantly higher risk of developing cardiovascular disease, diabetes, and neurocognitive impairment than the non-obese population. Thus, obesity may impact the health of HIV/AIDS patients as obesity may lead to the occurrence of non-AIDS-related comorbidities [15, 22, 24, 25].

Different INSTIs may cause different ranges of weight gain. Exploring the effect of INSTI on a patient’s body weight may contribute to better weight control in patients with HIV/AIDS. Therefore, in this study, we compared the effects of different INSTIs on body weight in HIV/AIDS patients to identify the one that causes the most significant weight gain in HIV/AIDS patients, using a network meta-analysis.

## Method

### Meta registration

This meta-analysis was reported according to the general guidelines outlined in the PRISMA (Preferred Reporting Items for Systematic Reviews and Meta-Analyses) statement. The study protocol has been registered on INPLASY PROTOCOL (registration number INPLASY2020120067).

### Literature search

PubMed, Embase, Cochrane Library, China National Knowledge Infrastructure (CNKI), Chinese Biomedical Literature Database (CBM), China Science and Technology Journal Database (VIP), and Wanfang databases were searched to obtain literature on INSTI in treating AIDS. The search time was from database establishment to October 15, 2020. The search languages were Chinese and English. The main search terms were "AIDS", "HIV", "Acquired Immunodeficiency Syndrome", "weight", "Raltegravir", "Elvitegravir", "Dolutegravir", "Bictegravir", "Integrase strand transfer inhibitor". The search formula is:((("Acquired Immunodeficiency Syndrome"[Mesh]) OR (((((((((((((((((Acquired Immuno-Deficiency Syndrome[Title/Abstract]) OR (Acquired Immuno Deficiency Syndrome[Title/Abstract])) OR (Acquired Immunodeficiency Syndromes[Title/Abstract])) OR (AIDS[Title/Abstract])) OR (Human Immunodeficiency Virus[Title/Abstract])) OR (Human T Cell Lymphotropic Virus Type III[Title/Abstract])) OR (Human T-Cell Lymphotropic Virus Type III[Title/Abstract])) OR (Human T-Cell Lymphotropic Virus Type III[Title/Abstract])) OR (Human T Cell Leukemia Virus Type III[Title/Abstract])) OR (LAV-HTLV-III[Title/Abstract])) OR (Lymphadenopathy-Associated Virus[Title/Abstract])) OR (Lymphadenopathy Associated Virus[Title/Abstract])) OR (Human T Lymphotropic Virus Type III[Title/Abstract])) OR (Human T Lymphotropic Virus Type III[Title/Abstract])) OR (AIDS Virus[Title/Abstract])) OR (AIDS Viruses[Title/Abstract])) OR (HTLV-III[Title/Abstract]))) AND ((((Raltegravir[Title/Abstract]) OR (Elvitegravir[Title/Abstract])) OR (Dolutegravir[Title/Abstract])) OR (Bictegravir[Title/Abstract]))) AND (weight[Title/Abstract]).

### Inclusion and exclusion criteria

Inclusion criteria: (1) HIV/AIDS patients with a definite diagnosis; (2) the ART uses INSTIs; (3) weight change before and after treatment was recorded.

Exclusion criteria: (1) no statistical analysis was performed or the relevant data were absent; (2) Duplicated publication of the same patient cohort; (3) The medications used in ART did not include DTG, BIC, RAL, or EVG; (4) Meta-analysis or review of the literature.

### Literature screening and data extraction

For the literatures with selective reporting results, we eliminated them. Due to the limitation of language, we only searched the published literatures. For the gray literatures, we did not include them in the study. The retrieved studies were screened by two researchers independently according to the inclusion and exclusion criteria and then cross-checked. Discrepancies in assessments were solved by consulting a third party. Later, two investigators extracted relevant data of the literature included, including first author, publication year, publication country, sample size, age, gender, and weight change.

### Literature quality evaluation

The included studies were cohort studies or randomized controlled trials. The quality of cohort studies was assessed using the Newcastle–Ottawa scale (NOS) scale, and the quality of randomized controlled trials was evaluated using the Jadad scoring scale. High-quality literature was defined as a Jadad score of (4–7). High-quality literature was defined as a NOS score of (5–9).

### Statistical methods

The data were analyzed using STATA 16.0. Measurement data were expressed as weighted mean difference (MD). Interval estimation was performed using a 95% confidence interval (CI) as an indicator of effect size. The node-splitting model was applied to analyze the direct comparison results and the indirect comparison results to observe the consistency. If there was no statistical difference (*p* > 0.05), the extracted data were subjected to network meta-analysis using the consensus model; if there was a statistical difference (*p* < 0.05), specific analysis was performed for items that showed inconsistency. After comparing the impacts of different INSTIs on weight gain, probability ranking plots were made to show the results.

## Results

### Process and results of literature retrieval

**Fig. 1:**
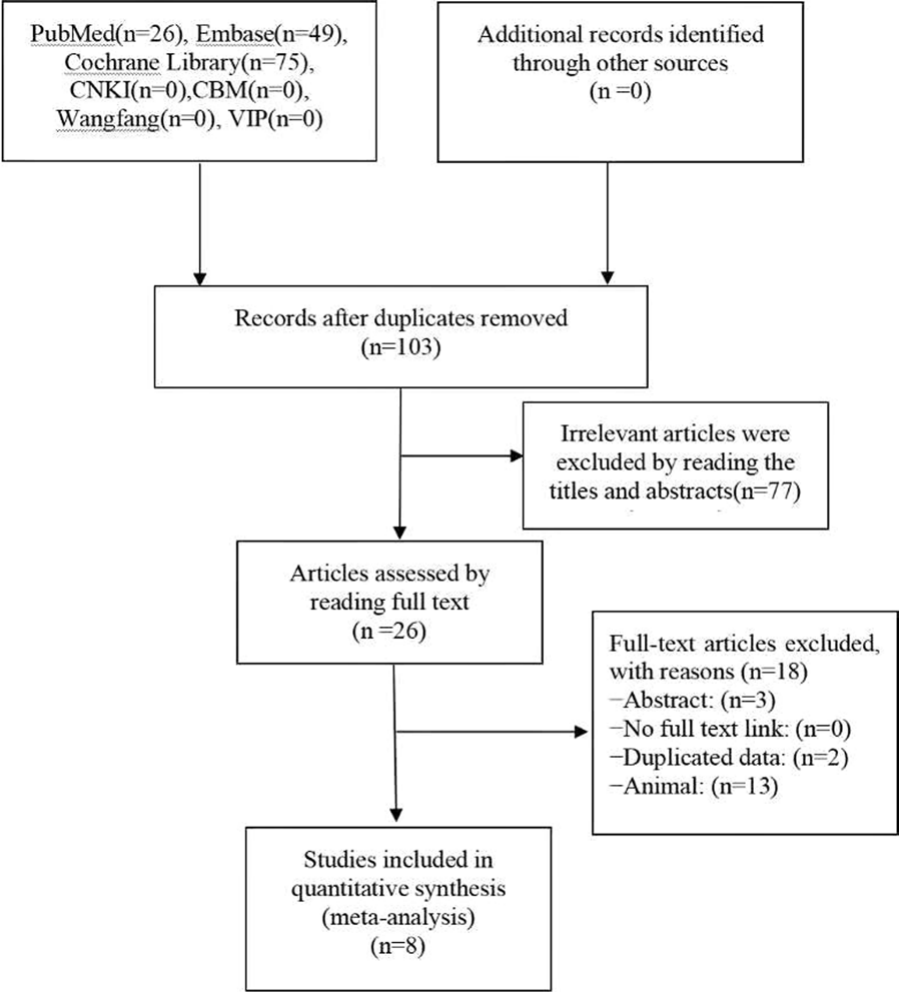
Literature search flow chart

A total of 150 relevant articles were selected during the initial search. All of which were published in English and involved DTG, RAL, EVG, and BIC in the ART interventions. According to the inclusion and exclusion criteria, 103 studies were considered eligible and retrieved for data extraction. After screening the titles and abstracts, 26 studies were kept. Then, the literature with abstract only, studies unavailable in full text, duplicated publications, and animal studies were excluded after reading the full text of the studies. Finally, eight studies [13, 14, 26–31] were included in this research (Fig. 1).

### Basic characteristics and quality evaluation of the included literature

Eight articles included consisted of three randomized controlled trials and five cohort studies, with a total of 11,339 patients. The basic characteristics and quality evaluation results of the included studies are shown in Table 1. The quality assessment of included studies showed that the Jadad scores of randomized controlled trials were all greater than 4 points (Additional file 1: Supplementary Table 1), and the NOS scores of cohort studies were all greater than 5 points (Additional file 2: Supplementary Table 2). Therefore, the overall quality of the studies included was high.

**Table 1:** Basic characteristics and quality evaluation of the included studies

Author	Year	Country	Type of study	Sex (M/F)	Ages	Sample sizes	Interventions	NOS/Jadad Scores
Leonardo Calza [26]	2019	Italy	Cohort study	138/58	43.1 ± 15.2	196	RAL	6
				115/59	41.6 ± 12.8	174	DTG	
				109/49	42.5 ± 13.6	158	EVG	
Peter [27]	2020	USA	Cohort study	917/164	44(33–52)	1081	RAL	7
				2058/257	34(27–45)	2315	EVG	
				1044/166	35(28–48)	1210	DTG	
Kassem Bourgi [28]	2020	USA	Cohort study	NA	NA	63	RAL	5
				NA	NA	153	EVG	
				NA	NA	135	DTG	
Kassem Bourgi [13]	2020	USA	Cohort study	1016/176	NA	1192	RAL	7
				795/131	NA	926	DTG	
				1842/226	NA	2068	EVG	
Sax [14]	2020	USA	RCT	565/74	37 ± 11.9	639	DTG	4
				434/67	38 ± 9.5	501	BIC	
				567/62	34 ± 10.8	629	EVG	
David A Wohl [29]	2019	USA	RCT	282/33	35(26–40)	315	DTG	4
				285/29	31(25–41)	314	BIC	
Stellbrink [30]	2019	Germany	RCT	280/40	33(27–46)	320	BIC	6
				288/37	34(27–46)	325	DTG	
Lake [31]	2020	USA	Cohort study	NA	NA	198	DTG	5
				NA	NA	204	EVG	
				NA	NA	289	RAL	

*NA* not available; *F* female; *M* male; *RCT* randomized controlled trial

### Evidence network

**Fig. 2:**
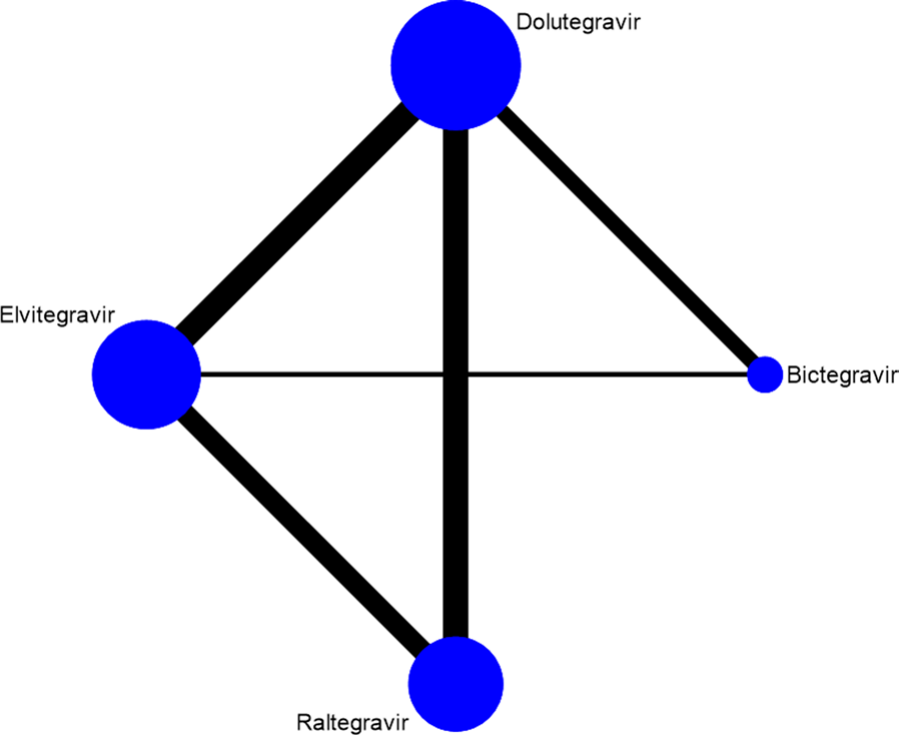
Network evidence diagram

Relationships among INSTIs were presented based on data from direct comparison. Each vertex on the relationship diagram represents an INSTI. The size of the vertex indicates the sample size. The line between the vertices represents the direct comparison between the two INSTIs. The boldness of the line is directly proportional to the number of studies of each pair of INSTIs. From Fig. 2, it can be found that there is direct or indirect evidence among the four types of DTG, RAL, EVG, and BIC. This diagram served as the basis for the network meta-analysis.

### Network meta-analysis

All studies included [13, 14, 26–31] reported weight changes in HIV/AIDS patients. The weight gain in patients who received DTG was significantly higher than that in patient who had EVG (MD = 1.13, 95% CI 0.18, 2.07). There was no significant difference in weight gain observed in comparisons between BIC, DTG, EVG and RAL (Table 2).

**Table 2:** Results of a network Meta-analysis of weight gain in patients with HIV/AIDS (MD, 95% CI)

BIC			
0.06 (− 1.15, 1.27)	DTG		
1.19 (− 0.22, 2.60)	1.13 (0.18, 2.07)	EVG	
0.80 (− 0.70, 2.29)	0.73 (− 0.22, 1.69)	− 0.39 (− 1.42, 0.63)	RAL

### Ranking of probability for weight gain for each drug

**Fig. 3:**
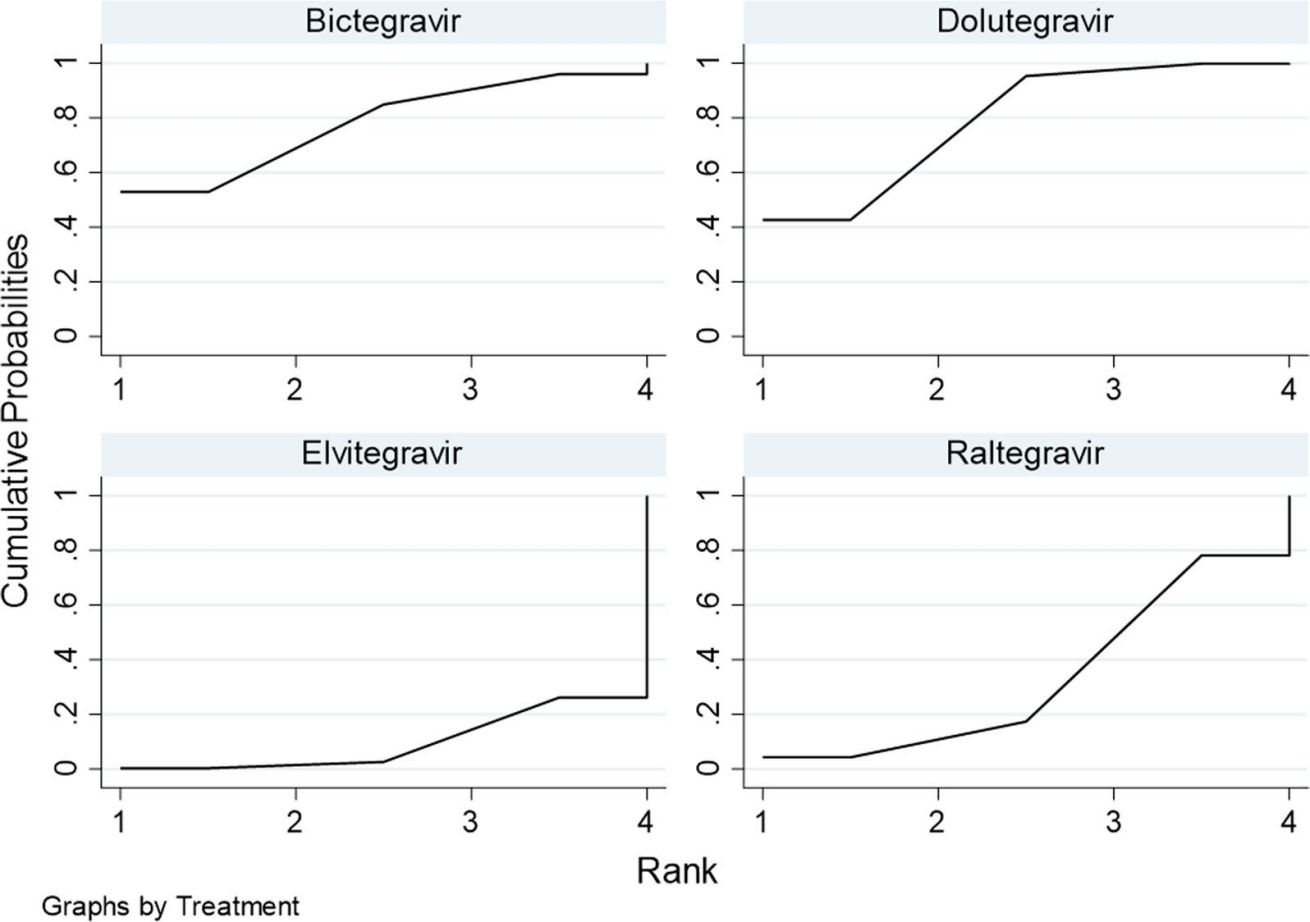
Probability ranking of each drug to increase body weight in HIV/AIDS patients (SUCRA)

The impacts of DTG, RAL, EVG, and BIC on body weight in HIV/AIDS patients are different. The cumulative ranked area under the curve (SUCRA) indicates the magnitude of the probability of each drug in causing significant weight gain. The greater the probability, the more likely a drug will lead to significant weight gain. The probability ranking order was: DTG (79.2%) > BIC (77.9%) > RAL (33.2%) > EVG (9.7%). This suggests that DTG may be the drug that causes the most significant weight gain in HIV/AIDS patients (Fig. 3).

### Consistency test

The inconsistency test result indicated no significant difference between direct and indirect comparisons (*p* > 0.05). Therefore, the results were analyzed using a consistency model. The results of node analysis showed that there was no significant difference in direct and indirect comparison between BIC versus DTG, BIC versus EVG, DTG versus EVG, DTG versus RAL, and DTG versus RAL (*p* > 0.05), indicating no local inconsistency was present (Table 3).

**Table 3:** Node analysis of direct and indirect comparisons among interventions

Category	Direct		Indirect		Difference		P
	Coef	95% CI	Coef	95% CI	Coef	95% CI	
BIC vs DTG	− 0.162	(− 1.47,1.15)	1.105	(− 3.40,5.61)	− 1.267	(− 5.96,3.43)	0.597
BIC vs EVG	− 0.76	(− 3.03,1.51)	− 1.504	(− 3.43,0.43)	0.744	(− 2.24,3.73)	0.625
DTG vs EVG	− 1.16	(− 2.19,-0.13)	− 0.752	(− 4.34,2.83)	− 0.408	(− 4.14,3.32)	0.831
DTG vs RAL	− 0.795	(− 1.82,0.23)	0.472	(− 4.11,5.05)	− 1.267	(− 5.96,3.43)	0.597
EVG vs RAL	0.463	(− 0.69,1.61)	− 0.113	(− 3.20,2.97)	0.576	(− 2.72,3.87)	0.732

*Coef* coefficient; *95% CI* 95% confidence interval

## Discussion

Our study is the first research to compare the differences in weight gain of patients receiving different INSTIs. The results of this network meta-analysis showed that DTG is associated with more significant weight gain than EVG. Meanwhile, the probability ranking revealed that DTG is the drug that causes the most significant weight gain in patients among all INSTIs. Weight gain is a commonly observed manifestation in HIV/AIDS patients after receiving ART [22]. The long-term health risk of obesity in HIV/AIDS patients remained unclear. Nevertheless, many studies have confirmed the risk for cardiovascular disease in patients with obesity [15, 22, 24, 25]. Therefore, obesity in HIV/AIDS patients should attract our attention.

The mechanisms by which different INSTIs contribute to weight gain have not yet been identified, and weight gain itself may be associated with multiple factors. A study [32] reported in vitro activity of DTG in inhibiting melanocortin four receptor (MC4R). MC4R plays a role in homeostasis, and its level of activity correlates with weight change. MC4R gene is shared between humans and mice. When the investigators knocked out the MC4R gene in mice, the mice demonstrated severe obesity [33]. Accordingly, after MC4R inhibition, caused by DTG, the patient's body weight increased significantly. Some scholars have suggested that the difference in weight gain may be related to the effect of INSTIs on adipocytes. A study [34] found that the concentrations of different antiretroviral drugs in adipose tissue are different. Comparing to other antiretroviral drugs, DTG and EVG are found in higher concentrations. An in vitro study [35] found that EVG impairs the metabolism of adipocytes, but RAL does not appear to damage adipocytes. Another hypothesis suggests that INSTIs might affect the gut microbiota in HIV/AIDS patients [36]. El Kamari et al. [37] found that fatty acid-binding protein level, a marker of intestinal integrity, can be used as an independent predictor of weight gain and visceral fat gain in HIV/AIDS patients. Despite various explanations and hypotheses established, the exact mechanisms leading to significant weight gain in HIV/AIDS patients remained unclear.

There might be alternative interpretations to the data obtained in this study. The increase in body weight may relate to successful inhibition of viral replication, control of inflammation, and reduction of energy expenditure at rest [38]. RAL and EVG are the first generation INSTIs, and DTG and BIC are the second generation INSTIs. DTG and BIC are superior to the first generation in terms of antiviral efficacy. RAL and EVG have a lower genetic barrier to resistance and, therefore, are more likely to induce drug resistance than DTG and BIC [39, 40]. Some studies found virological suppression rates of 83%, 85%, and 88% for EVG, RAL, DTG, respectively [41, 42]. The viral inhibition rate of DTG is higher than that of EVG and RAL, resulting in a low viral load in the patient and less energy expenditure at rest in the patient. This allows more energy to be stored in the body as fat.

In this study, we ranked RAL, EVG, DTG, and BIC by the probability of causing significant weight gain. According to the results, DTG was the INSTI that caused the most significant weight gain in patients with HIV/ADIS. BIC was the second on the ranking. Some studies have compared the virological inhibition rates of DTG and BIC, and the results showed that the efficacy of DTG was better than that of BIC [43]. Weight gain was higher in patients treated with DTG than in those treated with BIC, which further proved the link between low viral load and low resting state energy expenditure.

Weight gain has attracted the attention of the public increasingly. Because weight gain will increase the risk of non-AIDS-related diseases such as cardiovascular and cerebrovascular diseases in patients with HIV/AIDS. However, the risk of metabolic or cardiovascular diseases in HIV/AIDS patients cannot be predicted by the degree of obesity alone, as fat distribution is usually not included in the assessment of weight gain. Visceral fat or plasma level of fat/cholesterol is linked to the increased incidence of visceral or vascular diseases. Since the literature included in this study did not include data about peripheral or central obesity in HIV/AIDS patients, we could not evaluate the incidence of a certain type of obesity associated with INSTIs. Besides, whether some important metabolic parameters are correspondingly altered has not been effectively confirmed. It is expected that there will be subsequent studies on the health risk associated with fat gain in different regions of the body and abnormal metabolic parameters in HIV/AIDS patients, and such research may contribute to the development of the guideline for long-term health care of HIV/AIDS patients.

This study found that DTG may be the drug that causes the most significant weight gain in HIV/AIDS patients. The quality of this study is not as good as that of a randomized controlled trial due to the types of studies included in this research. This study had other limitations. The subjects in the included studies may have different basal body mass index, CD4^+^ T cell count, and dietary habits. The influence of these factors on weight change could not be excluded. The conclusion of this study is based on network meta-analysis, which has weaker strength of evidence comparing to the results generated by direct comparison. Further studies with rigorous design and large sample size are still needed to confirm the findings in this study.

## Conclusion

Based on data from the study included, DTG was found to have the greatest impact on weight gain in HIV/AIDS patients, followed by BIC. However, it is unclear whether INSTI-based regimens will lipohypertrophy (particularly an increase in visceral fat) or whether they increase the risk of cardiometabolic diseases. Therefore, future studies should focus on these unsolved issues and help to establish guidelines to take care of patients with HIV/AIDS effectively.

## Supplementary Information

**Additional file 1.** Jadad Quality Assessment.

**Additional file 2.** NOS Quality Assessment.

## Data Availability

All the data and materials are available from Pubmed, Cochrane Library, MEDLINE/EMBASE and Web of Science.

## Abbreviations

INSTI: Integrase strand transfer inhibitor
HIV: Human immunodeficiency virus
AIDS: Acquired immunodeficiency syndrome
CNKI: China National Knowledge Infrastructure
CBM: Chinese Biomedical Literature Database
DTG: Dolutegravir
EVG: Elvitegravir
BIC: Bictegravir
RAL: Raltegravir
ART: Antiretroviral therapy
PRISMA: Preferred Reporting Items for Systematic Reviews and Meta-Analyses
MD: Mean difference
95% CI: Confidence interval
PI: Protease inhibitor
NNRTI: Non-nucleoside reverse transcriptase inhibitor
EFV: Efavirenz
MC4R: Melanocortin four receptors
NA: Not available
F: Female
M: Male
RCT: Randomized controlled trial
Coef: Coefficient
Std.Err: Standard Error
