# Supplementary material for: Effects of different integrase strand transfer inhibitors on body weight in patients with HIV/AIDS: a network meta-analysis
Source: BMC Infect Dis. 2022 Feb 3;22:118. doi: 10.1186/s12879-022-07091-1 (PMC8811997; doi:10.1186/s12879-022-07091-1)
Supplement: Supplementary file 1 — Additional file 1. Jadad Quality Assessment. [file 12879_2022_7091_MOESM1_ESM.doc]

Supplementary Table 1 Jadad Quality Assessment

| study | year | Generation of random sequence | Randomization concealment | Blind method | Withdrawal and loss of follow-up | Total |
| --- | --- | --- | --- | --- | --- | --- |
| Sax PE^[14]^ | 2020 | 2 | 1 | 1 | 0 | 4 |
| David A Wohl^[29]^ | 2019 | 2 | 1 | 1 | 0 | 4 |
| Stellbrink^[30]^ | 2019 | 2 | 2 | 2 | 0 | 6 |
