# Supplementary material for: Effects of different integrase strand transfer inhibitors on body weight in patients with HIV/AIDS: a network meta-analysis
Source: BMC Infect Dis. 2022 Feb 3;22:118. doi: 10.1186/s12879-022-07091-1 (PMC8811997; doi:10.1186/s12879-022-07091-1)
Supplement: Supplementary file 2 — Additional file 2. NOS Quality Assessment. [file 12879_2022_7091_MOESM2_ESM.doc]

Supplementary Table 2 NOS Quality Assessment

|  |  | Selection |  |  |  |  | Exposure |  |  |  |
| --- | --- | --- | --- | --- | --- | --- | --- | --- | --- | --- |
| study | year | Adequate definition of cases | Representativeness of the cases | Selection of Controls | Definition of Controls | Comparability | Ascertainment of exposure | Same method of ascertainment for cases and controls | Non-Response rate | Total |
| Leonardo Calza^[26]^ | 2019 | ✮ | ✮ | ✮ | ✮ | ✮ | ✮ |  |  | 6 |
| Peter F^[27]^ | 2020 | ✮ | ✮ | ✮ | ✮ | ✮ ✮ | ✮ |  |  | 7 |
| Kassem Bourgi^[28]^ | 2020 | ✮ | ✮ | ✮ | ✮ | ✮ |  |  |  | 5 |
| Kassem Bourgi^[13]^ | 2020 | ✮ | ✮ | ✮ | ✮ | ✮ | ✮ |  | ✮ | 7 |
| Lake^[31]^ | 2020 | ✮ | ✮ | ✮ |  | ✮ | ✮ |  |  | 5 |
